# Nanoscale Pore Refinement and Hydration Control in Anhydrite-Modified Supersulfated Cement: Role of Calcination-Induced Crystal Phase Transition

**DOI:** 10.3390/nano15181432

**Published:** 2025-09-18

**Authors:** Zeyuan Hu, Cheng Zhang, Yi Wan, Rui Ma, Chunping Gu, Xu Yang, Jianjun Dong, Dong Cui

**Affiliations:** 1 School of Safety Science and Engineering, Nanjing University of Science and Technology, Nanjing 210094, China; zeyuan_hu@163.com (Z.H.); chen-zhang@njust.edu.cn (C.Z.); 2 School of Physics, Nanjing University of Science and Technology, Nanjing 210094, China; wany@njust.edu.cn; 3 School of Materials and Chemical Engineering, Anhui Jianzhu University, Hefei 230601, China; marui@ahjzu.edu.cn; 4 School of Civil Engineering, Zhejiang University of Technology, Hangzhou 310014, China; guchunping@zjut.edu.cn; 5 Nanjing KENTOP Civil Engineering Co., Ltd., Nanjing 210041, China; xu_yang0917@163.com

**Keywords:** supersulfated cement, anhydrite modification, nanoporous structure, hydration control, phase transition

## Abstract

Nanostructural optimization is key to enhancing the performance of low-carbon cements. Supersulfated cement (SSC) is an eco-friendly, low-carbon cement primarily composed of blast furnace slag and calcium sulfate. This study investigates the effects of two types of crystalline anhydrite on the hydration degree and strength of SSC. The experiment used III ${CaSO}_{4}$ (high solubility) and II-U ${CaSO}_{4}$ (low solubility) as sulfate activators, evaluating the mechanical properties of anhydrite produced at different calcination temperatures through an analysis of pore structure, phase composition, reaction degree of mineral powder, and hydration heat. The results indicate that II-U anhydrite enhances slag hydration, reduces pore size, and significantly improves the compressive strength of SSC. This improvement is attributed to its impact on slag hydration: it reduces gypsum consumption rate, delays ettringite formation, promotes gel product formation, decreases the volume ratio of ettringite to calcium silicate hydrate (C-S-H) gel, fills pores, and decreases porosity. This study reveals the influence of calcined dihydrate gypsum phase changes on the macroscopic properties of SSC and the microstructure of hydration, elucidating the hydration mechanism of anhydrite-based SSC. This work provides a nanomaterial-based strategy for SSC design via crystal phase engineering.

## 1. Introduction

Traditional Portland cement, a primary component of conventional concrete, is manufactured using mineral resources as raw materials and fossil fuels as the main energy source in high-temperature kilns. This process results in substantial carbon dioxide emissions, significantly contributing to the intensification of the greenhouse effect and environmental degradation [1,2,3]. Consequently, the development of environmentally sustainable cementitious materials has become a critical objective in the field of construction materials [4]. SSC [5] has emerged as a promising alternative due to its low carbon footprint and sustainable characteristics. SSC offers several advantages, including minimal clinker content, high utilization of industrial solid waste, strong resistance to chemical attack, low heat of hydration, and high late-stage compressive strength, which have attracted increasing attention in recent years [6].

SSC is primarily composed of ground granulated blast furnace slag (GGBFS, typically over 80%), calcium sulfate (10–20%), and a small amount of alkaline activator. The hydration mechanism of SSC mainly involves the reaction of calcium aluminate phases in the slag with calcium hydroxide (CH), calcium sulfate, and water. The alkaline activator first reacts to produce CH and alkali metal hydroxides, which facilitate the dissolution of the slag surface. Subsequently, dissolved aluminum, calcium, and silicon ions react with calcium sulfate to form ettringite and C-S-H [7,8]. Ettringite is primarily responsible for early compressive strength development (within 7 days), while C-S-H contributes to the strength gain at later stages [9]. Additionally, phases such as AFm (monosulfate) and hydrotalcite may also form during hydration.

The early hydration of SSC is largely controlled by the dissolution rate of the slag and the subsequent precipitation of hydration products. Therefore, to improve early-age strength, many studies have focused on accelerating the dissolution rate of slag to promote the formation of hydration products. The dissolution behavior is influenced by several factors, including the pH of the pore solution, the ${Al}_{2}O_{3}$ content in slag, curing conditions, and the type and dosage of activators [10].

Among these factors, the pore solution pH plays a vital role in regulating hydration kinetics. The formation and characteristics of SSC hydration products depend strongly on whether the pore solution pH falls within an optimal range. Zhou, Peng, and colleagues [11,12,13] investigated the influence of various weak acids (e.g., sodium gluconate, sodium tartrate, sodium citrate, and sodium lactate) on the hydration microstructure, strength, and freeze–thaw resistance of SSC. They proposed a pH-dependent hydration regulation mechanism. Masoudi and Angulski et al. [14,15] explored the effect of slag alumina content on SSC hydration activity, finding that slags with less than 13 wt% ${Al}_{2}O_{3}$ were unsuitable for SSC production due to insufficient reactivity. Chen et al. [16] studied the use of nano-silica (NS) to enhance SSC hydration by facilitating the interaction between silicate and aluminate species. Their findings showed that NS improved slag reactivity, refined the microstructure, and significantly enhanced early-age compressive strength. This was attributed to delayed ettringite formation, promoted precipitation of Calcium-(Aluminum)-Silicate-Hydrate (C-(A)-S-H), and reduced micro-pore formation.

While increasing slag dissolution rate has proven effective for enhancing early hydration, another strategy involves optimizing the composition of raw materials—particularly by using specialized forms of calcium sulfate and appropriate levels of alkaline activators. Rubert et al. [17] found that varying calcium sulfate content (10–20%) had little impact on compressive strength and heat evolution, but did influence C-S-H formation, with lower calcium sulfate or activator contents promoting C-S-H development. Gracioli et al. [18] studied anhydrite produced by calcining phosphogypsum (PG) at 350 °C and 650 °C, and analyzed the effects of its solubility on SSC performance. Hooton [19] emphasized that anhydrite solubility plays a critical role in SSC hydration, and that early curing at elevated temperatures (38 °C or 50 °C) reduces solubility, thereby hindering the slag reaction and decreasing strength. Liu et al. [20] investigated PG modified via lime neutralization and calcination, demonstrating improved hydration behavior and enhanced compressive strength in SSC, along with a refined pore structure.

Based on the above background, in this paper, the analytical pure gypsum was put into a calcination dish, added to Muffle furnace, and calcined at 350 °C and 600 °C [8], respectively, for 2 h at constant temperature to produce III ${CaSO}_{4}$ and II-U ${CaSO}_{4}$ [20]. Two types of anhydrite were added to the SSC system as sulfate activators in various experiments to investigate the impact of different types of anhydrite on the hydration reaction of the SSC system.

This study aims to provide a novel approach for the preparation of SSC and offers valuable insights for its application in engineering practice. By optimizing the interaction between SSC and anhydrite, the mechanical properties of SSC can be effectively enhanced to meet the requirements of diverse construction scenarios. However, the nanoscale mechanisms behind anhydrite crystal phases (e.g., pore structure evolution, C-S-H gel nucleation) remain unclear. This study deciphers the nanostructure–property relationships in anhydrite-modified SSC, offering a crystal phase design principle for eco-friendly cement nanomaterials.

## 2. Materials and Methods

### 2.1. Raw Materials

**Figure 1:**
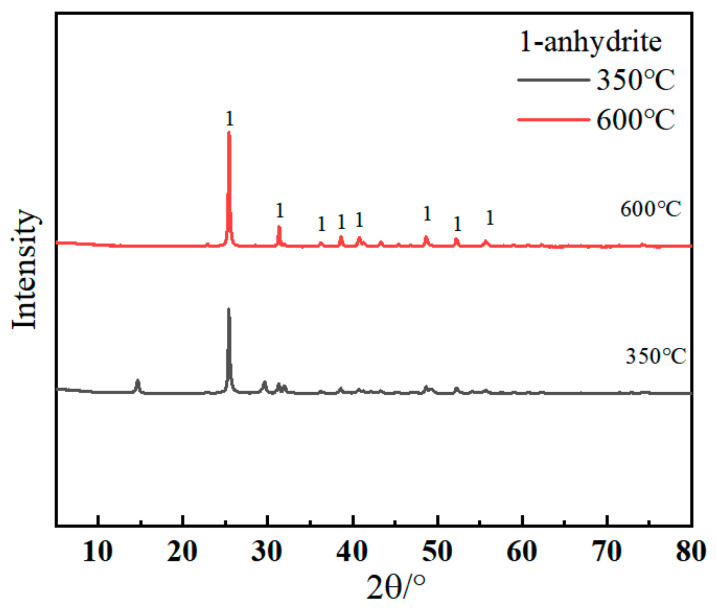
XRD analyses of modified gypsum.

The main composition of SSC is ground granular blast furnace mineral powder (Qingdao, China), alkaline activator as the reference cement (Linyi, China), gypsum (Shanghai, China), anhydrite (by analytical pure dihydrate gypsum calcined at 350 °C and 600 °C [8], respectively, for 2 h at constant temperature to produce III ${CaSO}_{4}$ and II-U ${CaSO}_{4}$, as shown in Figure 1). All fine aggregate sand used in the mortar test is standard sand (Xiamen ASIO Standard Sand Same companies), which meets the ISO standard. The chemical composition of reference cement and mineral powder is shown in Table 1.

### 2.2. Sample Preparation

**Figure 2:**
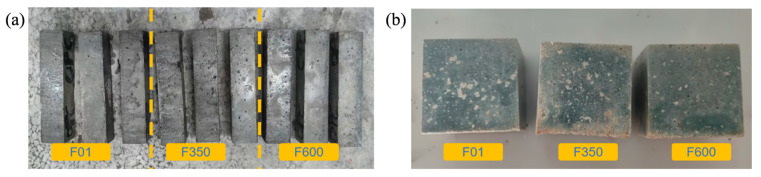
Sample preparation of supersulfated cement: (**a**) mortar samples and (**b**) paste samples.

The sample was prepared in accordance with the Chinese standard GB/T 17671-2021 [21]. The formulations of the mixtures investigated in this study are detailed in Table 2. The water–binder ratio (w/b) for all paste samples is 0.4, while the water–binder ratio for all mortar samples is set at 0.5 to ensure fluidity and prevent bleeding, as shown in Figure 2. Following the specified standard curing environment (RH > 95%, 20 ± 2 °C) and the standard curing time (3 d, 7 d, 28 d), the paste sample is immersed in isopropyl alcohol for 24 h and then transferred to fresh isopropyl alcohol. Subsequently, the sample is immersed in fresh isopropyl alcohol for 4 days to halt hydration, after which it is dried in a vacuum drying oven at 40 °C for 3 days. Finally, the sample is sealed in a bag and stored in a vacuum drying oven.

### 2.3. Methods

#### 2.3.1. Test of Mechanical Properties of Mortar Samples

According to the “Test Method for Cement Mortar Strength” (GB/T 17671-2021), bending and compressive tests are conducted on mortar test specimens that have been maintained in a standard curing laboratory at the ages of 3 days, 7 days, and 28 days. The unit of measurement is MPa, and the numerical variance represents the error, accurate to 0.1 MPa.

#### 2.3.2. Heat of Hydration

The experiment was conducted using an eight-channel microcalorimeter (New Castle, DE, USA) with the method of external stirring isothermal calorimetry. Initially, the instrument was balanced at 25 °C to calibrate the baseline. Subsequently, the pure paste was prepared according to the experimental ratio, quickly weighed, and then dropped into the ampoule and placed in the channel. Deionized water was chosen as the reference and was added to the sample simultaneously. The experimental data was then recorded continuously until 72 h of hydration.

#### 2.3.3. X-Ray Diffractometer (XRD)

The XRD test was conducted using the D8 Advance diffractometer from Bruker, Germany. Prior to the test, the powder sample and the internal standard corundum (20% of the total mass) were placed in an agate mortar and ground with ethyl alcohol for 30 min to ensure thorough mixing. The sample was then screened through a 200-mesh sieve. The scanning range was set at 5° to 80° (2θ), with a scanning rate of 10°/min. Subsequently, the Rietveld method was employed to refine the structure, resulting in more accurate results. The phase content was calculated using TOPAS 4.2 software, and quantitative results for each phase were obtained through the Rietveld method refinement.

#### 2.3.4. TG-DTG

The American TA thermogravimetric analyzer was employed to quantitatively calculate the content of ettringite, gel chemically combined water, and gypsum in each set of paste samples hydrated for a specific duration. Following the cessation of hydration with isopropyl alcohol, the samples were dried in a vacuum oven at 40 °C for 72 h and then ground with a mortar for no less than 10 min. The ground samples were subsequently tested after passing through a 200-mesh sieve. The test was conducted under the protection of inert gas, with a gas flow rate of 50 mL/min, a test temperature range of 30–1000 °C, and a heating rate of 10 °C/min.

#### 2.3.5. C-(A)-S-H Gel Quantification

The thermogravimetric analysis results were analyzed. The mass loss between 50 °C and 500 °C is attributed to the C-(A)-S-H gel, AFt, and gypsum binding water [22,23]. Typically, one mole of AFt corresponds to 32 moles of water, while one mole of gypsum corresponds to 2 moles of water molecules. Therefore, the mass fraction of combined water for AFt and gypsum (ω AFt) can be calculated using Equation (1):

(1) $$\;\omega_{AFt}=\frac{M_{H}\times 32}{M_{A}},\omega_{Gyp}=\frac{M_{H}\times 2}{M_{G}}$$

where $M_{H}$ is the mass fraction of $H_{2}O$, $M_{A}$ is the mass fraction of AFt, and $M_{G}$ is the mass fraction of gypsum (the results were obtained by quantitative XRD analysis). The combined water content of AFt and gypsum (${BW}_{AFt}$ and ${BW}_{Gyp}$) can be calculated by Equation (2):

(2) $$\;{BW}_{AFt}=M_{A}\times \omega_{AFt},{BW}_{Gyp}=M_{G}\times \omega_{Gyp}$$

By subtracting the combined water content of gypsum and AFt from the total combined water content (${BW}_{Total}$), the binding water content of C-(A)-S-H gel can be obtained (${BW}_{C-\left(A\right)-S-H}$):

(3) $$\;{{BW}_{C-\left(A\right)-S-H}=BW}_{Total}-{BW}_{AFt}-{BW}_{Gyp}$$

#### 2.3.6. Selective Dissolution of EDTA Mineral Powder

The degree of hydration of mineral powder is determined in accordance with the selective dissolution method [24,25] outlined in GB/T 12960-2019. This method leverages the solubility of cement, gypsum, and hydration products within the mineral-cement hydration system, as well as the insolubility of unhydrated mineral powder. To prepare the triethanolamine solution with a 1/3 volume fraction, sodium hydroxide was used to adjust the solubility, along with an EDTA solution at a concentration of 150 mmol/L. Subsequently, 50 mL of the EDTA solution, 10 mL of the triethanolamine solution, and 120 mL of water were sequentially added to a 250 mL beaker. The pH of the solution was then adjusted to 11.60 ± 0.05 using NaOH solution, as indicated by the pH meter. A powder sample weighing 0.3000 ± 0.0100 g, which had ceased hydration and was ground after passing through a 200-mesh sieve, was stirred and dissolved under magnetic stirring for 30 min. Following this, air was immediately pumped through a glass sand core funnel containing a layer of 0.45 μm filter paper, and the insoluble matter was washed with water and aqueous ethanol a total of 10 times. The insoluble matter was subsequently placed in a drying oven at 105 ± 5 °C until reaching a constant weight, and then weighed using an analytical balance with an accuracy of 0.0001 g to determine the mass of the insoluble matter.

#### 2.3.7. ICP-OES

The ion concentration of the pore solution was measured using the Spectro Bluesop tester manufactured by Spectroblue in Germany. The pore solution of pH, once tested, is sealed and stored at a temperature of 3–5 °C to prevent any deterioration resulting from environmental changes or temperature fluctuations. When testing is required, the solution is retrieved and analyzed for calcium ions, sulfur ions, silicon ions, and aluminum ions. Given that different ions have different concentrations, silicon and aluminum ions are initially tested in the original solution to ensure accuracy. Subsequently, calcium and sulfur ions are tested after acidification and dilution with a mass fraction ranging from 0.1% to 10% to facilitate precise measurement of their concentrations.

#### 2.3.8. Pore Structure Analysis (1H NMR)

A cement paste cylinder with dimensions of Φ20 mm × 20 mm was prepared, and the transverse relaxation time $T_{2}$ distribution of the sample was measured using the CPMG pulse sequence. The pore distribution was estimated based on Equation (4).

(4) $$\;\frac{1}{T_{2}}=\rho_{2}{\left(\frac{S}{V}\right)}_{pore}=F_{s}\times \frac{\rho_{2}}{r}$$

where $T_{2}$ is the transverse relaxation time; $\rho_{2}$ is the surface relaxation rate, where the surface relaxation efficiency of $\rho_{2}$ is 10 μm/s [26,27]. ${\left(\frac{S}{V}\right)}_{pore}$ is the specific surface area of the pore; $F_{s}$ is a geometrical factor, which is 2.0 for cylindrical pores [28,29]. r is the pore size.

## 3. Results and Discussion

### 3.1. Compressive Strength

**Figure 3:**
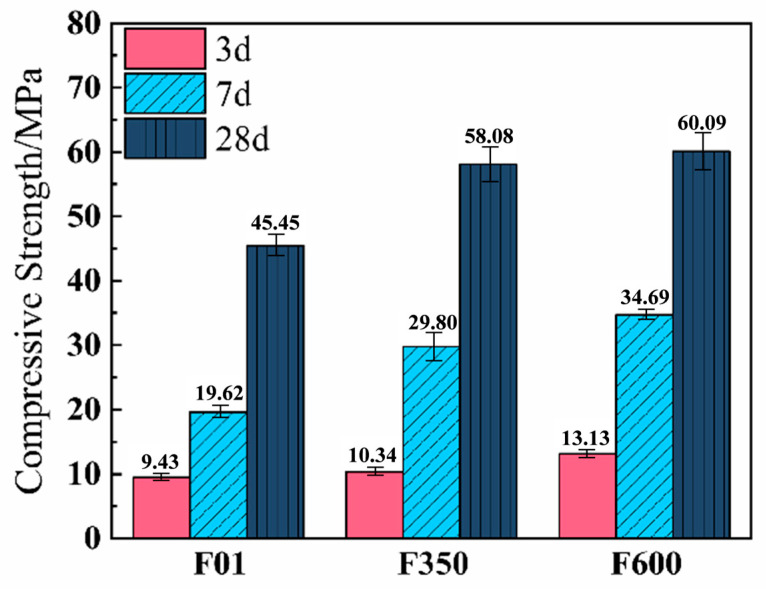
Compressive strength development of SSC.

The compressive strength results, as shown in Figure 3, compare the performance of the F350 and F600 systems. Both systems exhibited similar 28-day strength; however, their early-age strength development differed significantly. The early strength of the III-anhydrite system (F350), produced by low-temperature calcination, was relatively low. Nevertheless, its strength increased by 188.2% after 7 days, surpassing the 133.1% increase observed in the F600 system. This indicates that while F350 developed strength more slowly at early ages, it exhibited a more rapid gain at later stages—suggesting that the later-age strength development is closely related to the distinct crystalline properties of the anhydrite types. Unlike II-U anhydrite, III-anhydrite possesses a metastable crystal structure and higher solubility within the SSC system, following a “dissolution–crystallization” mechanism [30]. When the concentrations of Ca^2+^ and SO_4_^2−^ ions in solution exceed the solubility threshold of dihydrate gypsum, the anhydrite undergoes a phase transformation and precipitates as dihydrate gypsum, driven by the crystallization potential. This process consumes a significant amount of free water in the system, thereby accelerating the setting process. In a supersaturated environment, the precipitation of dihydrate gypsum is enhanced, which occurs concurrently with ettringite formation. As a result, part of the early strength is contributed by gypsum crystallization. The subsequent rapid strength gain in the F350 system can be attributed to the declining solubility of III-anhydrite with increasing curing temperature—though it remains higher than that of other sulfate sources. This solubility behavior promotes the continued generation of dihydrate gypsum through the solution–crystallization mechanism. The F350 system retains abundant Ca^2+^ and SO_4_^2−^ ions in solution, and the dihydrate gypsum formed from III-anhydrite exhibits a higher dissolution rate compared to that formed from II-U anhydrite. Consequently, the F350 mix facilitates more robust ettringite formation, reduces system water demand, decreases porosity, and enhances compressive strength.

**Figure 4:**
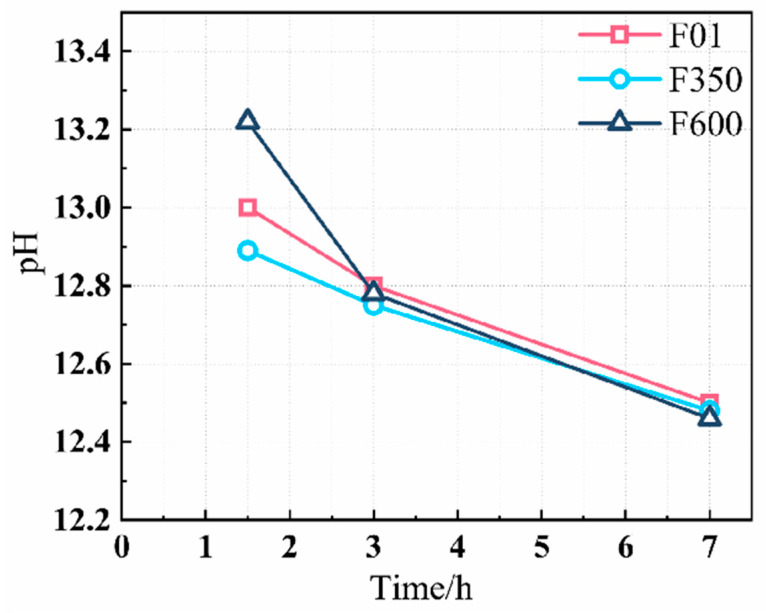
PH value of early hydration reaction.

As shown in Figure 4, the blank group results further illustrate that F600 begins to dissolve early upon water contact, resulting in a relatively high pH level. This elevated pH enhances the initial dissolution of slag, promoting early hydration. Moreover, III-anhydrite is characterized by a dense crystal structure and high specific surface area. These properties improve particle dispersion and packing within the system, minimizing inter-particle voids, increasing contact between particles, and enhancing particle-to-particle bonding.

Such improved particle packing is conducive to hydration reactions and facilitates the growth of hydration products. Additionally, the fine particle size of III-anhydrite contributes to pore filling, thereby reducing total porosity and concentrating pore size distribution within the mesoporous range (0–50 nm).

Overall, the calcined anhydrite systems demonstrated significant strength improvements over the unmodified gypsum at all ages. The presence of anhydrite alters the microstructure of the mortar matrix, improving both the compactness and homogeneity of the SSC system. This enhancement ultimately leads to improved compressive strength, meeting the minimum requirements specified in the European standard for supersulfated cement (EN 15743:2010 [31]). These findings clearly demonstrate that anhydrite, when used as a sulfate source, outperforms dihydrate gypsum in terms of strength development.

### 3.2. Hydration Process of Cement Paste

**Figure 5:**
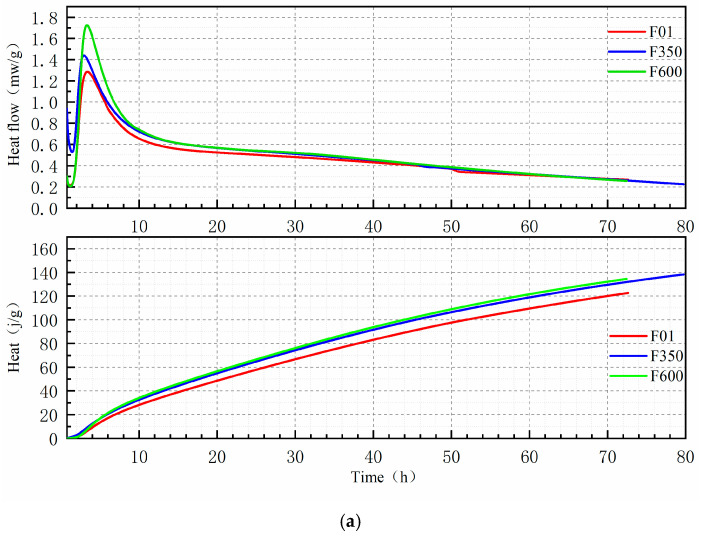

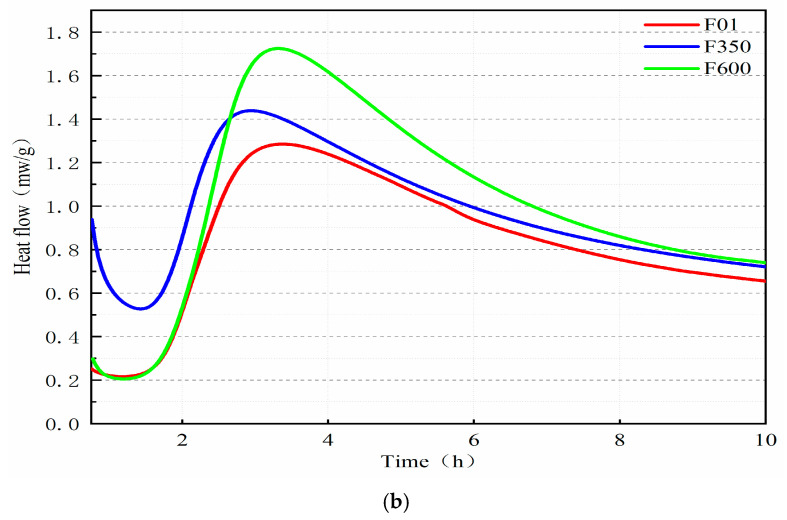
(**a**) The hydration heat of the SSC containing different sulfates. (**b**) The hydration heat of the SSC containing different sulfates (0.75~10 h).

The thermal evolution of SSC hydration is generally comparable to that of ordinary Portland cement (OPC). As illustrated in Figure 5, the hydration process can be divided into five distinct stages: pre-induction (I), induction (II), acceleration (III), deceleration (IV), and stability (V). In this figure, the actual reaction time begins at 45 min, as the initial mass peak—caused by external stirring during sample preparation—is excluded. The time interval from 0.45 to 1.5 h corresponds to the induction period, 1.5 to 3 h represents the acceleration period, 3 to 11 h defines the deceleration phase, and the period beyond 11 h marks the stable stage. During hydration, the gradual accumulation of hydration products increases the density of the system, thereby impeding water diffusion and limiting further hydration. As diffusion resistance rises, the reaction rate declines and eventually stabilizes. Consequently, the cumulative heat release increases at a slower rate during the later stages.

From Figure 5b, it is evident that the first exothermic peak of F350—incorporating III-anhydrite—occurs at 3 h, which is earlier than that observed in F01 and F600. This suggests that F01 and F600 exhibit lower early-age solubility, slower slag dissolution rates, and limited ionic release, all of which contribute to sluggish hydration kinetics and reduced early heat evolution. In contrast, III-anhydrite in F350 acts as an efficient sulfate activator due to its high solubility, providing a rapid and abundant supply of Ca^2+^ and SO_4_^2−^ ions. As the system dissolves continuously, the alkalinity increases. Under the influence of OH^−^ ions, the surface of the slag is disrupted, facilitating the dissolution of reactive ${SiO}_{2}$ and ${Al}_{2}O_{3}$ phases. This promotes the early formation of ettringite and other hydration products. As a result, the induction period is shortened, the exothermic peak of the acceleration period appears earlier, and the overall heat release is enhanced. These findings confirm that the addition of III-anhydrite accelerates the early hydration reactions and enhances the thermal output of the system.

A comparison between F600 and F01 reveals that although their induction periods are similar, F600 exhibits a higher exothermic peak during the acceleration phase. This indicates that hydration reactions between the mineral powder and II-U anhydrite are more complete in F600. While the solubility of II-U anhydrite is comparable to that of F01, its larger specific surface area and more uniform dispersion in the slag matrix improve its reactivity. During dissolution, Ca^2+^ and SO_4_^2−^ ions from II-U anhydrite interact readily with the silica tetrahedra in the slag. Over time, this enhances the hydration process, promoting greater formation of ettringite and C-S-H gel. Consequently, the heat evolution increases, and the total cumulative heat release after 3 days exceeds that of both F350 and F01. This observation is consistent with the XRD analysis results.

### 3.3. Quantitative Phase Analysis of Hydration Products

**Figure 6:**
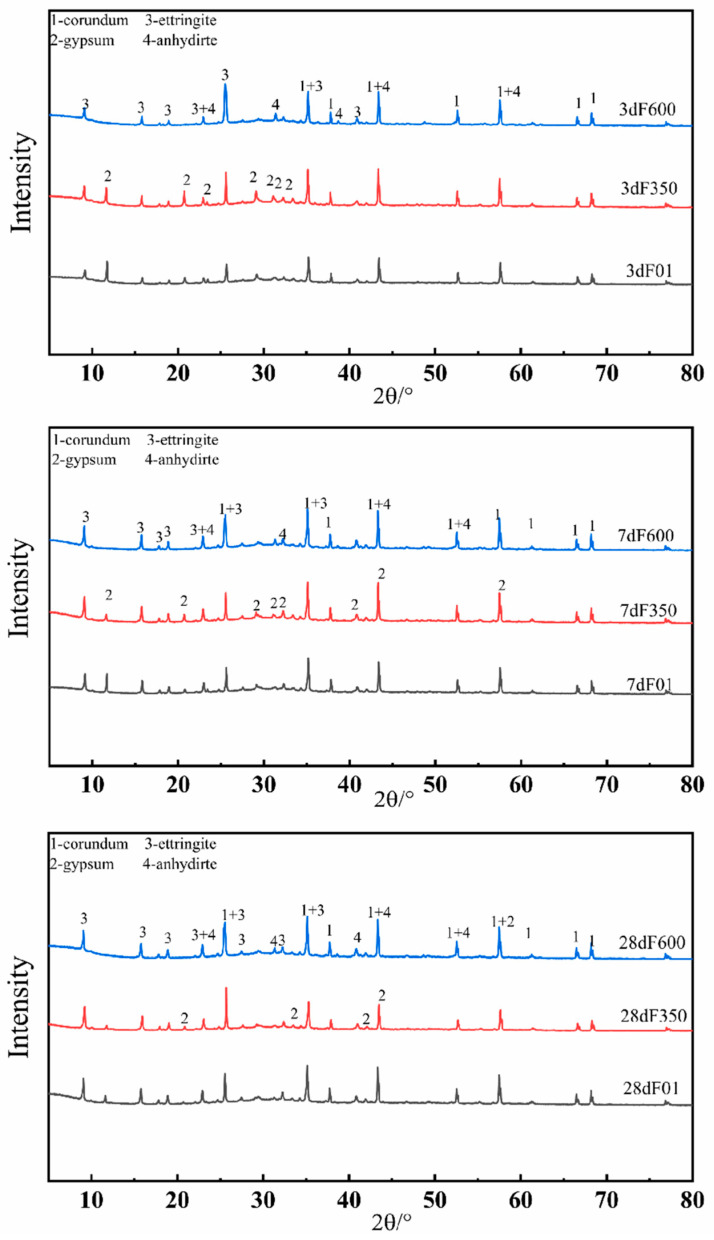
XRD analyses of F01, F350 and F600 after 3, 7 and 28 days of hydration.

The XRD analysis was conducted to characterize the formation of hydration products in the SSC samples, and the results are displayed in Figure 6. The main phases identified in the SSC spectrum include unreacted mineral phase, hydrated product phase, and intermediate product phase. These phase evolutions not only reflect the hydration kinetics of SSC but also provide experimental evidence for the activation mechanism of internal sulfate crystals.

The primary hydration products of SSC consist of AFt (ettringite) and C-S-H gels, with the characteristic peaks of AFt being clearly visible on the XRD pattern. A comparative analysis reveals that the highest peak of ettringite in the samples mixed with III anhydrite and II-U anhydrite as sulfate activator is notably greater than that of the control group F01. Quantitative analysis results from Table 3 indicate that the unreacted gypsum content of F350 is the highest at 3 days. As confirmed by the quantitative analysis, F600 produced the highest amounts of both AFt (13.48%) and C-S-H (15.04%) at 3 days, which highlights its superior nucleation effect compared with F01 and F350. This is further supported by the slower consumption of II-U anhydrite (from 7.10% at 3 days to 2.40% at 28 days, a reduction of 66.7%), compared with gypsum in F01 (77.4%) and F350 (80.5%). By contrast, F350 shows a different hydration pathway. At 28 days, it contains the highest amount of AFt (26.07%) but the lowest amount of C-S-H (13.14%). Although its compressive strength still reaches 58.8 MPa, this indicates that F350 tends to rely more on massive AFt formation rather than gel production, differing fundamentally from the C-S-H–dominated mechanism of F600. However, as the hydration age increases, the consumption rate of gypsum accelerates, leading to a decrease in characteristic peak intensity and an increase in the production of ettringite with age. This trend suggests an enhanced degree of hydration reaction in F350. Notably, In the early stages of F350, III anhydrite undergoes a hydration reaction, releasing ${Ca}^{2+}$ and ${SO}_{4}^{2-}$. Simultaneously, according to the principle of dissolution–crystallization, gypsum is produced. Secondary gypsum serves as an intermediate product to weaken its compressive strength. On the other hand, F600, incorporating II-U anhydrite, features a stable structure, small crystal particles, and a large specific surface area. These fine particles act as “nano-seeds”, providing numerous nucleation sites for hydration products [32,33]. This strategy is similar to that reported in recent studies where nano-seeds were used to enhance the early strength of blended cements. The “nano-seed” effect enhances particle distribution within the system and fills cracks alongside hydration products, ultimately reducing pores and improving compressive strength.

The volume ratio of AFt/C-S-H and the morphology distribution of hydration products are crucial factors impacting SSC. Early compressive strength increases with the rise in AFt mass fraction. Compared with the early F01, F350 exhibits higher compressive strength due to a larger proportion of AFt mass, coupled with the generation of dihydrate gypsum through the principle of “dissolution-crystallization”, effectively shortening the condensation time. However, the hydration products in F600 surpass those of F01, resulting in smaller system pores, which positively impacts the compressive strength, exceeding that of F01 and F350. In the later stages, as C-S-H gel products play a dominant role in cement hardening, F600 exhibits higher levels of gel products compared to F01, with a smaller volume ratio of AFt/C-S-H than F01, leading to increased hydration product quantities and stronger macroscopic mechanical properties. This is attributed to the lower consumption rate of anhydrite in the SSC compared to gypsum, thereby delaying the growth rate of ettringite, enhancing the degree of hydration reaction of mineral powder, promoting gel product output, filling pores, reducing porosity, and consequently improving the compressive strength of the SSC system.

### 3.4. Thermogravimetry (TG)

The hydration products of the SSC system can be quantitatively analyzed using thermogravimetric analysis technology. During the heating process, ettringite and C-S-H gel lose their binding water, allowing the hydration products to be reflected by the water loss. The intensity of diffraction peaks in the XRD pattern can be used for quantitative analysis of the amorphous phase, but there may be relative errors. Conversely, for the crystalline phase, absolute errors are observed. To accurately quantify the content of each phase, TG and XRD results can be cross-verified.

**Figure 7:**
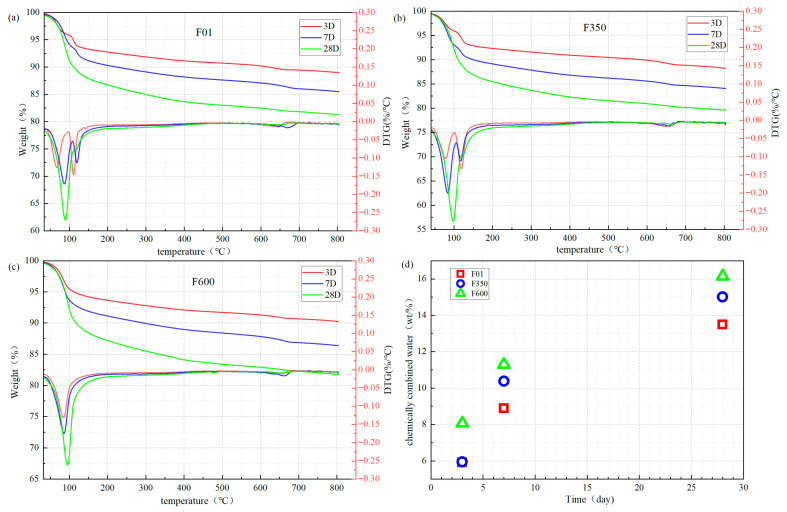
TG-DTG curve of sample ((**a**) F01, (**b**) F350 and (**c**) F600) and (**d**) the chemical bound water mass ratio of hydration products.

In the TG-DTG curve, the content of chemically bound water can be calculated based on the DTG result, reflecting the total amount of the two hydration products. The main water loss peaks of hydration products are concentrated in the range of 50 °C to 200 °C. In Figure 7, the first peak observed at 50 °C to 90 °C is generally attributed to the dehydration of C-S-H (or C-(A)-S-H) and ettringite [34]. Additionally, in the SSC system, the “third hydrate aluminate” (TAH) [35] combined with C-S-H gel will decompose in the temperature range of 70 °C to 90 °C. Moreover, the peak observed in the 100–150 °C range is attributed to the dehydration process of gypsum (${CaSO}_{4}\cdot 2H_{2}O$), during which the compound loses water molecules and transforms into anhydrite (${CaSO}_{4}$). The decarbonization of ${CaCO}_{3}$ takes place in the temperature range of 600–800 °C.

In order to calculate the content of chemically bound water in the hydration products more accurately, this paper adopts the peak-splitting method [36] to integrate the samples. The starting point of the steep drop of the first water loss peak in the ground was selected for analysis, and the DTG results at 50–500 °C were integrated. The maximum of the integral of the second peak at 100–200 °C was taken as the bound water content of gypsum, and the absolute difference in the two integral results was taken as the bound water content of hydration products.

The combined water content of gypsum and the combined water content of hydration products can be obtained by integrating the DTG results in different temperature ranges. Furthermore, when combined with the EDTA method, the degree of dissolution of mineral powder in the cement hydration process of the three samples can be understood. Through observation and analysis, compared with the blank group F01, the early incorporation of III anhydrite and II-U anhydrite promoted the development of low Ca/Si ratio C-S-H gel with a needle-like morphology [37]. These high-aspect-ratio C-S-H nanofibers interweave to form a three-dimensional nanonetwork, enhancing the cohesion and density of the later hydration gel and thereby significantly improving later-age strength and elasticity [38]. Together, TG, XRD and EDTA results confirm that the more complete hydration in sample F350 at 28 days results in stronger DTG peaks and wider peak widths—consistent with a denser, nanostructured C-S-H network.

### 3.5. Analysis of EDTA Mineral Dissolution and Hydration Products

#### 3.5.1. EDTA

According to the XRD results, the addition of anhydrite promotes the formation of ettringite and C-S-H gel in a high mineral powder system. The reaction degree of the mineral powder can be determined through EDTA tests conducted on samples of different ages, allowing the investigation of the hydration degree of each sample. The influence of different types of anhydrite on the growth rate of the hydration degree of the mineral powder in the SSC system is analyzed.

**Figure 8:**
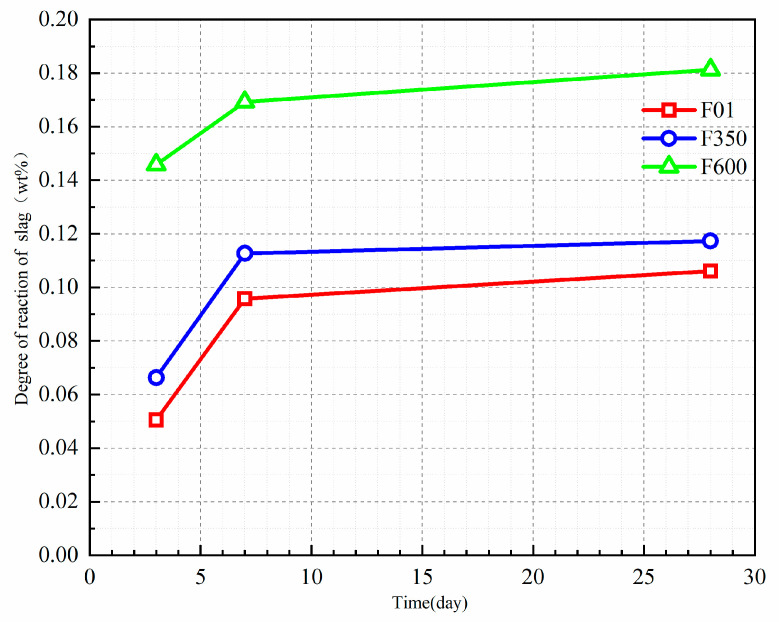
Reaction degree of EDTA mineral powder.

As shown in Figure 8, the addition of gypsum exhibits a significant growth rate in the hydration degree at early ages, while the reaction degree of the mineral powder gradually slows down with increased age. The inclusion of II-U anhydrite enhances the production of gel and promotes the hydration of the mineral powder. In both the SSC systems containing III anhydrite and II-U anhydrite, under the same mixing ratio, the addition of anhydrite as a sulfate activator increases the likelihood of the dissolution reaction of the mineral powder. This, in turn, improves the reaction degree between aluminum, silicon ions, CH, and sulfate ions, leading to the generation of more hydration products. Overall, this enhanced reaction degree contributes to the improvement of the compressive strength on a macroscopic scale.

#### 3.5.2. Analysis of Hydration Products

In order to investigate the promoting effect of different types of anhydrite on the hydration products of SSC, the mass fraction of hydration products at different ages in each sample group was analyzed and processed using EDTA and chemical combination water results to determine the content of AFt (ettringite) and C-S-H gel.

**Figure 9:**
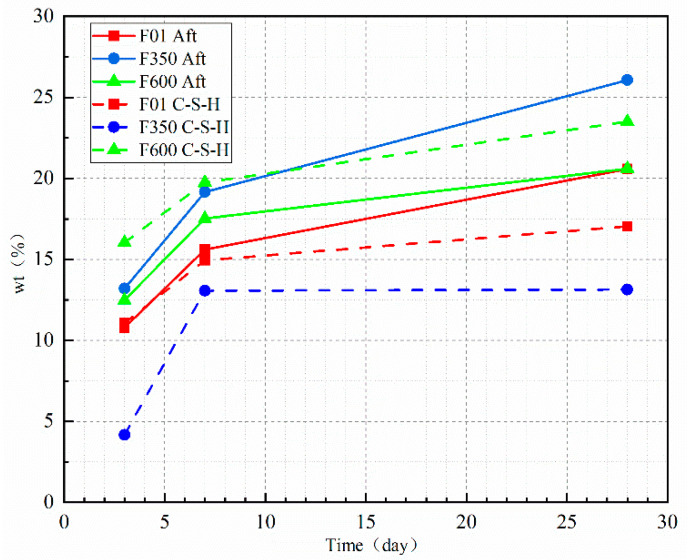
Phase distribution of hydration products.

The results in Figure 9 indicate that when gypsum is present, the content of ettringite in F01 is lower compared to anhydrite-based SSC. However, the gel content ratio is higher in F01 than in F350. This suggests that during the hydration of dihydrate gypsum, due to the rapid consumption of sulfate ions, the free solution fills the pores and promotes the generation of gel products. On the other hand, F350, with III anhydrite characteristics, creates a stable environment suitable for the growth of ettringite by reducing the pH of the system. Despite the reduction in gel product, the growth of ettringite is promoted, resulting in pore filling and improved compressive strength.

When II-U anhydrite is used as a sulfate activator, it increases the pH early on, promoting the dissolution of the mineral powder. The small crystal particle size and large contact area of the anhydrite particles facilitate the growth of ettringite. Additionally, the gradual increase in gel product and slower consumption of anhydrite delay the growth rate of ettringite. Through these combined effects, porosity is reduced, compressive strength is increased, and the structure becomes more stable.

In summary, the choice of different types of anhydrite influences the hydration products of SSC differently, affecting factors such as ettringite formation, gel product generation, pore filling, compressive strength, and structural stability.

### 3.6. Changes in Ion Concentration of ICP

**Figure 10:**
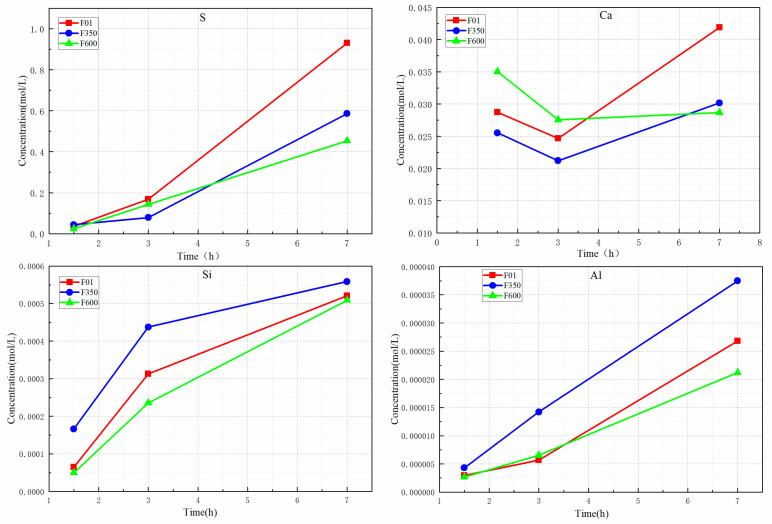
Different ions concentration of ICP (S, Ca, Si, Al).

The liquid phase collected 7 h before the reaction was subjected to pH testing, and ion concentrations were measured at various time points to analyze the influence of different types of gypsum on the ion concentration of the pore solution. The concentrations of calcium, silicon, sulfur, and aluminum ions in samples from each group were analyzed, as shown in Figure 10. ICP-OES was used to record the changes in the concentration of main ions in the pore solution of all samples during hydration.

For calcium ions, the addition of II-U anhydrite increases the concentration of Ca ions. Between 1.5 h and 3 h, it is observed that F600 consumes a large amount of Ca ions when it reaches the first exothermal peak. During the deceleration period, ion precipitation is hindered, and the consumption rate maintains a balance with the production rate. A similar trend is observed for F350. Compared with F01, although fewer Ca ions are decomposed in the induction period, more Ca ions are consumed in the hydration process, leading to the generation of more hydration products and higher compressive strength.

For silicon (Si) ions, the key observation is that the dissolution rate of mineral powder into the system exceeds the ion consumption rate generated by the hydration product gel. This leads to the filling of pores with Si ions during the hydration acceleration period, with F350 exhibiting the highest concentration. In terms of calcium (Ca) ions, it is evident that during the formation of gel products, the consumption rate is lower than the dissolution rate, indicating that the dissolution of gypsum hinders the hydration process at this stage, which is in line with the XRD results. After 3 h, the ion concentration of F600 is the lowest. Consequently, adding II-U anhydrite to the SSC system can enhance the reactivity of mineral powder and facilitate the production of C-S-H gel products.

The consumption rate of Al phase ions is mainly attributed to ettringite and C-(A)-S-H gel. From 1.5 h to 7 h, F600 and F01 show an increasing trend, indicating that the Al consumption rate is lower than the dissolution rate of mineral powder. This is due to the high solubility of II-U anhydrite, which promotes the reaction degree of mineral powder, precipitates a large amount of Al, and facilitates the formation of hydration products. III anhydrite in the sample F350, due to their high solubility, undergo rapid reactions during hydration. They dissolve and precipitate dihydrate gypsum, leading to reduced gypsum consumption. As a result, the rate of ${Ca}^{2+}$ consumption in the hydration reaction cannot keep up with the dissolution rate of the mineral powder, thereby inhibiting the generation of hydration products.

As for S ions, they are primarily transformed into sulfate ions during the induction period of 1.5 h to 3 h. Gypsum dissolution releases heat, promoting sulfate ion precipitation and generating hydration products in the pores. After 3 h, the ion concentration increases, and gypsum dissolves in large quantities, causing the delay in ettringite formation and contributing to sustainable development and growth of intensity.

**Figure 11:**
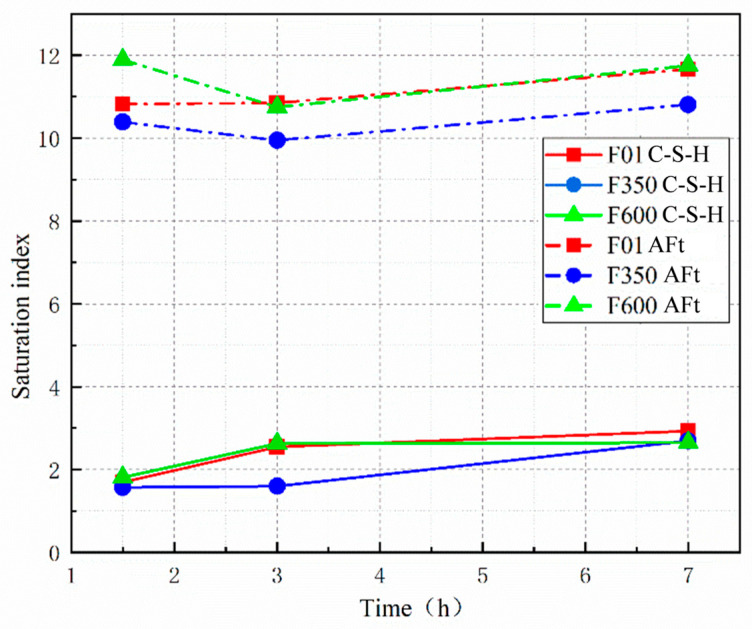
Saturation index of hydration products.

Through Gems thermodynamic simulation of the saturation index of hydration products, it can be seen from Figure 11 that compared to F01, the AFt saturation index in SSC systems with different crystal types of hard gypsum initially decreases and then increases. This indicates that the driving force for the early precipitation of ettringite first decreases and then increases, gradually increasing during the hydration decay period. Additionally, the saturation index of C-S-H gel in the calcium salt system generally shows an upward trend, indicating an increased driving force for C-S-H gel precipitation during the hydration process, making gel formation easier. The saturation index of AFt and C-S-H gel in II-U anhydrite is higher than that of the other two groups. The simulation results align with the trends observed in the heat of hydration and thermogravimetric analysis. This suggests that adjusting the concentration ratio of the pore solution with II-U anhydrite can improve the precipitation efficiency of hydration products and significantly enhance the compressive strength of the system. The slow dissolution characteristic of II-U anhydrite (Figure 4) maintains the supersaturation of Ca^2+^/SO_4_^2−^ in the nanopore solution (Figure 11), prolongs the C-S-H nucleation period, and forms a more uniform nano-gel, compared to the local defects caused by the rapid crystallization of F350.

### 3.7. Pore Structure Analysis of 1H-NMR

**Figure 12:**
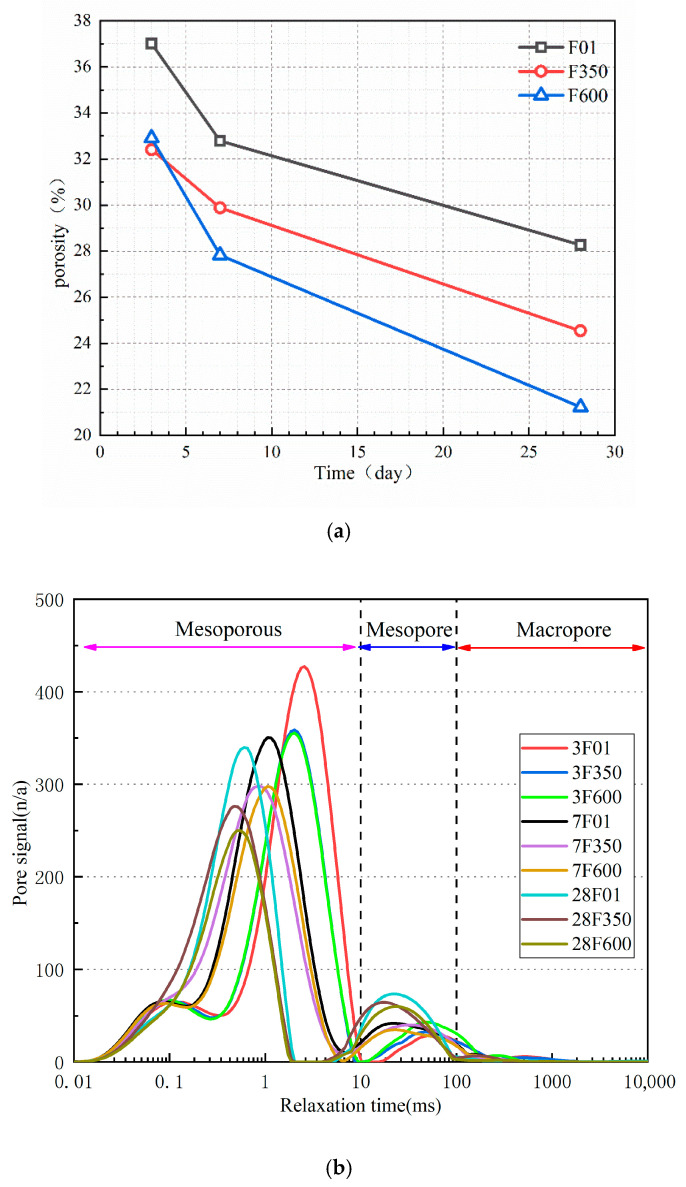

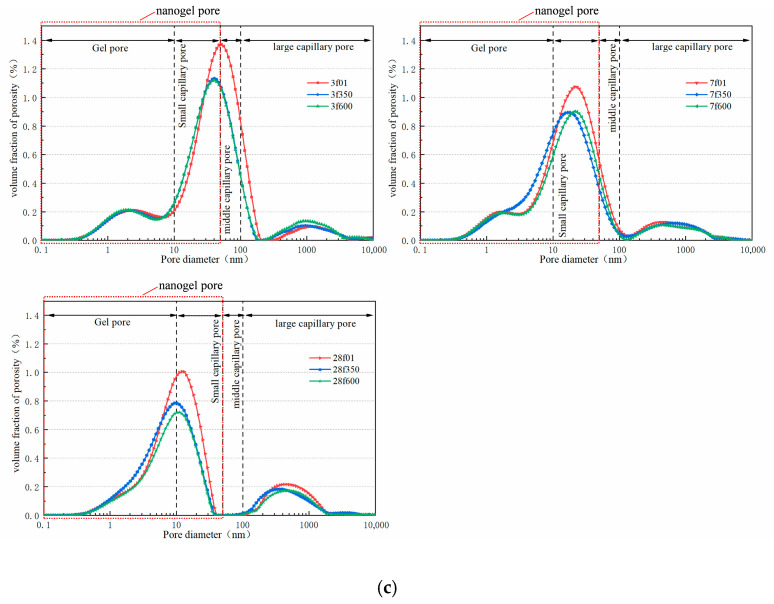
(**a**) Sample porosity (F01, F350 and F600). (**b**) $T_{2}$ patterns of relaxation time of samples with different hydration times. (**c**) Pore size distribution (3 d, 7 d, 28 d).

When the cement samples were immersed in vacuum water for 24 h, the relaxation time distribution of all samples exhibited a main peak, accompanied by a few weak secondary peaks. The relaxation time of the main peak in the NMR $T_{2}$ spectrum was primarily between 2–10 ms. As the curing time progressed, the relaxation peak gradually shifted to the left, towards shorter relaxation times. Besides the change in the position of the relaxation peak, the pore signal strength of the relaxation peak also decreased gradually with the extension of the curing time. This decrease in pore signal strength is due to the long relaxation time of free water and the short relaxation time of chemically bound water (less than 0.01 ms), which cannot be effectively detected by low-field NMR. Additionally, as shown in Figure 12b, the relaxation time of the main peak of F01 is 6.05 ms at 3 days, while the relaxation time of the main peak of F350 and F600 is similar, less than 5 ms. The transverse relaxation time of the main peak reflects the state of hydrogen protons in most components of the cement-based material, corresponding to the internal structure of the material and thus indicating the compressive strength of the sample. There is a negative correlation between the transverse relaxation time corresponding to the main peak and the compressive strength. Specifically, the smaller the transverse relaxation time, the greater the compressive strength. The transverse relaxation time of water in the pore is linearly related to the pore size—in other words, the shorter the relaxation time, the smaller the pore size. With increasing age, the signal strength of F600 and F350 decreases, and the transverse relaxation time decreases compared with the control group. This suggests that part of the free water in the anhydrite cement reacts with the cement, gradually transforming into CH, thus creating an alkaline environment. This stimulates the formation of combined water between gypsum and mineral powder, generating hydration products to fill the pores. Consequently, the main peak of the $T_{2}$ spectrum gradually shifts towards shorter relaxation times, leading to a gradual decrease in pore signal strength.

The porosity of the samples, calculated by integrating the total signal area of the T_2_ relaxation spectra shown in Figure 12b, is presented in Figure 12a. Analyzing the porosity of SSC-based cement in Figure 12a, it is evident that the porosity of samples in each group rapidly decreases during the hydration reaction, with F600 showing the fastest reduction from 32.91% to 21.23%. This reduction in porosity correlates negatively with compressive strength, indicating that smaller pores result in greater compressive strength. Moreover, when considering XRD and TG data, the small particle size and large specific surface area of II-U anhydrite interact with hydration products during the early hydration process, forming matrix effects. This alignment of molecules, along with the collaboration of mineral powder particles and hydration products, fills cracks, reduces pores, and enhances strength. Specifically, the nanocrystalline grains of II-U anhydrite (with a crystallite size of ~27 nm, as estimated from the XRD analysis) and the C-S-H gel (1–100 nm [39]) synergistically form a dense nanocomposite structure. This nano-filler effect not only fills the interstitial voids but also promotes a more refined pore network, thereby reducing pore connectivity and enhancing the material’s durability [32].

The evolution of pore size distribution is presented in Figure 12c. As hydration age increases, the main peak in the T_2_ spectrum shifts from the capillary pore range to the gel pore range. This indicates a transformation in the pore structure: large capillary pores diminish in proportion, while gel pores become more prevalent. At 3 days, the dominant peak in all groups resides in the capillary pore range. The peak maximum for F01 is centered around 50 nm, while those for F350 and F600 are shifted toward the smaller capillary range. Over time, the pore size distribution curves of all groups shift leftward by approximately 20–50 nm. The peak area corresponding to larger pores decreases significantly, with F600 showing the smallest peak area and F01 the largest. This suggests that the incorporation of anhydrous gypsum as a sulfate activator improves the pore structure of SSC at the nanoscale. The hydration products generated fill capillary and intermediate pores, leading to a refined pore structure and a substantial reduction in porosity by 28 days [40].

## 4. Conclusions

Through phase analysis, liquid phase analysis, and mechanical properties analysis of each group of SSC samples, the mechanism for improving the hardening process of SSC under different anhydrite systems was investigated. The findings are as follows:

(1) Compared to the control group (F01), both F350 and F600, containing different types of anhydrite gypsum, exhibited significantly higher early and late compressive strengths, reaching nearly 60 MPa at 28 days, in contrast to 45 MPa for F01. This enhancement is primarily attributed to the modification of the SSC’s nanoscale pore structure by anhydrite gypsum, which led to an overall reduction in porosity. In particular, intermediate and large pores were effectively filled by hydration products, contributing to the improved mechanical strength. Specifically, the nanoscale particles of II-U anhydrite gypsum, characterized by high specific surface area, filled the capillary pores and converted those larger than 50 nm into finer gel pores smaller than 20 nm (Figure 12c), thereby enhancing strength through nanoscale densification.
(2) The hydration reaction process under different types of gypsum content exhibits relative similarity. F01 rapidly dissolves dihydrate gypsum in the early stages, but the reaction is later restricted due to insufficient gypsum, resulting in the lowest total heat release of hydration. F350 utilizes III anhydrite as the activator, which features high solubility. According to the principle of solution–crystallization, rapid generation of secondary gypsum occurs. During the transformation process, the system loses a significant amount of free water, leading to reduced setting time, reduced pH, increased ettringite, decreased gel formation, and promoted hydration reaction. The II-U anhydrite in F600 possesses relatively small crystal form and large specific surface area, increasing the contact area between particles and promoting the combination of particles in the system. Furthermore, the system’s high pH facilitates the dissolution of mineral powder, promotes the hydration reaction, and further increases the total heat release of hydration.
(3) The influence of anhydrite on the phase composition of the supersulfated system includes promoting the formation of hydration products and increasing the degree of mineral powder reaction. For the two kinds of anhydrite with different crystal forms, II-U anhydrite has the most noticeable promoting effect on mineral powder. The hydration products of the two types tend to form in different directions, with the hydration products of III anhydrite being mainly AFt, while II-U anhydrite primarily forms C-S-H gel. Moreover, II-U anhydrite induces the formation of highly cross-linked nanoscale C-S-H gel, whereas III anhydrite tends to generate micron-scale ettringite bundles, which reduces the toughness and increases the risk of brittle failure.
(4) The nanocrystalline anhydrite (II-U type) acts as a functional nanofiller and hydration modulator, demonstrating the potential of phase-engineered nanomaterials in sustainable construction.

## Figures and Tables

**Table 1:** Main chemical composition of raw materials (wt%).

	CaO	${\mathrm{Al}}_{2}O_{3}$	$\mathrm{Si}O_{2}$	MgO	$SO_{3}$	${\mathrm{Fe}}_{2}O_{3}$	LOI
OPC	64.14	4.12	18.53	1.75	3.34	2.76	5.36
GGBS	28.15	21.23	32.22	11.82	2.51	2.61	1.46

**Table 2:** Mixture proportion in this study (wt%).

Sample	GGBS	Clinker (OPC)	CaSO_4_•2H_2_O	III CaSO_4_	II-U CaSO_4_	Water–Binder Ratio (w/b)	
						Paste	Mortar
F01	84	1	15	0	0	0.4	0.5
F350	84	1	0	15	0	0.4	0.5
F600	84	1	0	0	15	0.4	0.5

**Table 3:** The hydration product in the specimen (wt%).

Specimen	Calcite	Ettringite	Gypsum	Bassanite	Anhydrite	C-S-H	AFt/C-S-H
3F01	0.47	10.79	3.90	--	--	11.08	0.97
3F350	0.53	13.20	9.23	0.13	0.34	4.18	3.16
3F600	0.46	13.48	0.27	0.21	7.10	15.04	0.90
7F01	0.57	15.61	2.07	--	--	14.94	1.05
7F350	0.70	19.16	2.81	0.23	0.26	13.07	1.47
7F600	0.63	17.52	0.22	0.38	3.54	19.75	0.89
28F01	0.62	20.58	0.88	--	--	17.04	1.21
28F350	0.51	26.07	1.80	0.17	0.12	13.14	1.98
28F600	0.56	21.60	0.30	0.27	2.40	23.51	0.88

## Data Availability

Data generated in this study are presented in the article.
